# Case Report: Anti-interferon-γ autoantibodies in an adolescent with disseminated *Talaromyces marneffei* and mycobacterial co-infections

**DOI:** 10.3389/fped.2025.1552469

**Published:** 2025-02-27

**Authors:** Bingkun Li, Tiantian Li, Qihua Huang, Nanfang Mo, Xiaojuan He, Zhiwen Jiang, Xiuying Li, Xiaolu Huang, Xinyu Zhang, Cunwei Cao

**Affiliations:** ^1^ Department of Dermatology and Venereology, The First Affiliated Hospital of Guangxi Medical University, Nanning, China; ^2^ Guangxi Scientific and Technological Innovation Cooperation Base of Mycosis Prevention and Control, Nanning, China

**Keywords:** anti-interferon-γ autoantibodies, *Talaromyces marneffei*, nontuberculous mycobacteria, adolescents, immunodeficiency syndrome

## Abstract

**Background:**

Anti-interferon-γ autoantibodies (AIGAs) are associated with adult-onset immunodeficiency syndrome, which makes individuals susceptible to intracellular pathogen infections. However, AIGAs are rarely reported in adolescents.

**Case presentation:**

We report a 13-year-old Chinese boy who presented with fever, cough, and enlarged cervical lymph nodes. Blood cultures yielded *Mycobacterium abscessus*, and *Talaromyces marneffei* (TM) was cultured from pericardial effusion. Whole exome sequencing revealed no pathogenic variants. Notably, high levels of neutralizing AIGAs were detected in the patient's serum. After receiving treatment for *Mycobacterium abscessus* and antifungal therapy for TM, the patient showed significant improvement. However, at the 19-month follow-up, the patient developed a *Mycobacterium asiaticum* infection.

**Conclusion:**

This case highlights the importance of screening for AIGAs in pediatric patients with disseminated TM or NTM infections. Prolonged treatment and continuous follow-up remains crucial for managing pediatric patients with AIGAs.

## 1 Introduction

Anti-cytokine autoantibodies are increasingly recognized as causes of susceptibility to severe infections and immunologic conditions. They affect cytokine biology by disrupting signaling pathways or altering their half-life in circulation. These include autoantibodies against interleukin-6 in staphylococcal disease, interleukin-17 in chronic mucocutaneous candidiasis, granulocyte–macrophage colony-stimulating factor in cryptococcosis or nocardiosis, type I interferons in severe COVID-19 pneumonia, varicella-zoster virus and adverse reactions to the live attenuated viral vaccine against yellow fever, interleukin-23 in Aspergillus, Coccidioides and Pneumocystis, and environmental mycobacteria infections (1, 2).

Interleukin-12 (IL-12) is a potent activator of the NK cells and T cells, which, in turn, secrete interferon-γ (IFN-γ) (3). IFN-γ is a crucial pro-inﬂammatory cytokine that binds to the IFN-γ receptor on phagocytes, enhancing their ability to eliminate intracellular pathogens and promoting the production of IL-12 (4). The IL-12/IFN-γ-related Inborn Errors of Immunity (IEIs) in children, such as those with Mendelian Susceptibility to Mycobacterial Disease (MSMD), render individuals highly susceptible to severe mycobacterial infections and salmonellosis, highlighting the critical role of the IL-12/IFN-γ axis in defending intracellular pathogens (5). Neutralizing anti-IFN-γ autoantibodies (AIGAs) cause an adult-onset immunodeficiency, which has been classified as category X of IUIS classification of IEI, also referred to as IEI phenocopies (6–8). Similar to pediatric patients with IL-12/IFN-γ-related IEI, patients with AIGAs are vulnerable to a specific range of opportunistic intracellullar pathogens, including nontuberculous mycobacteria (NTM), *Talaromyces marneffei* (TM), *Salmonella spp.*, varicella-zoster virus (8, 9). More than 600 patients with AIGAs have been diagnosed, the majority of whom are adults of Asian descent. However, only two pediatric cases have been reported (8). Here, we present the case of a 13-year-old boy with AIGAs who developed disseminated TM and multiple NTM infections within a 19-month period with the aim of enhancing clinicians' awareness of the disease.

## 2 Case report

**Figure 1:**
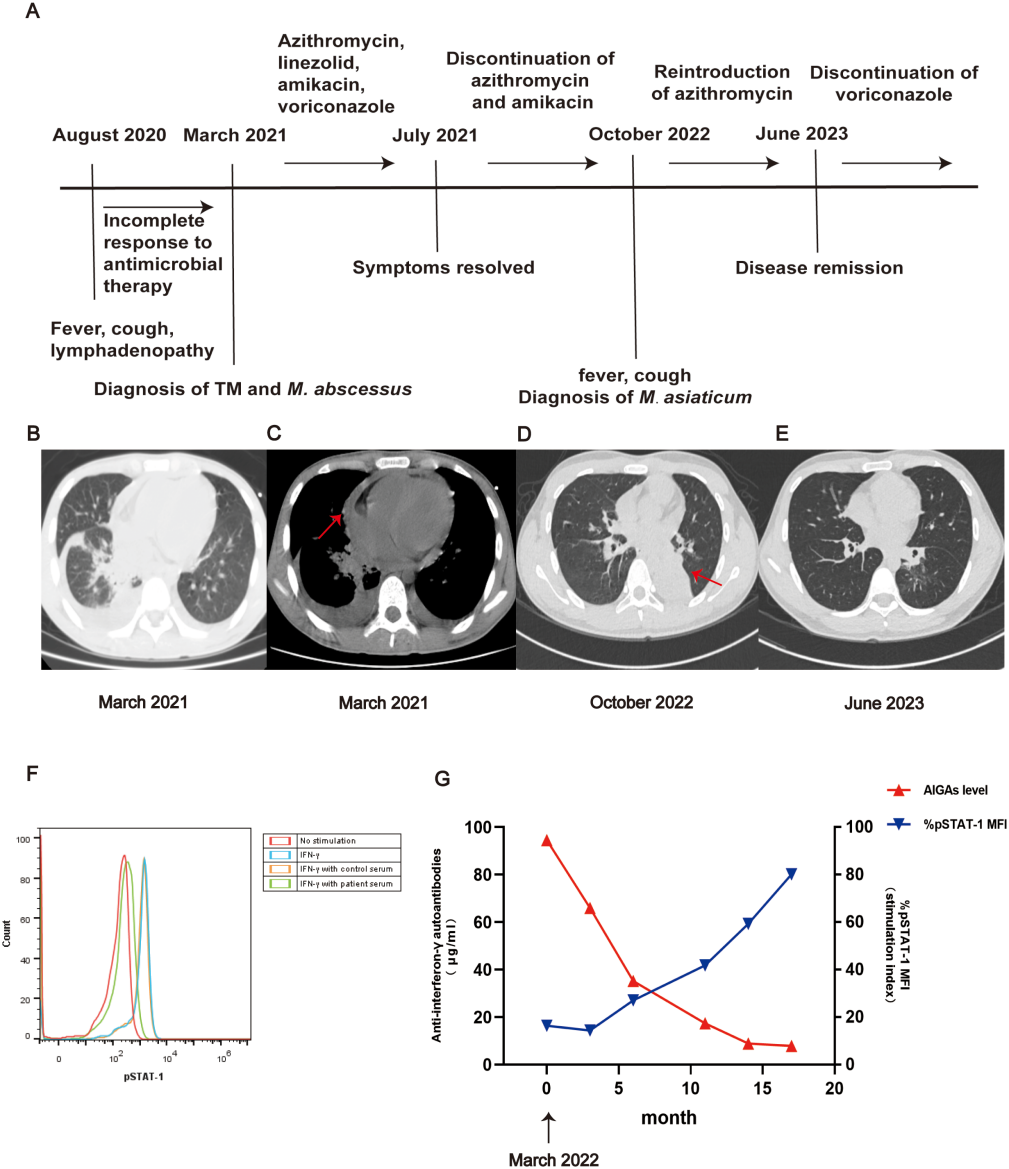
Clinical course of the case presentation **(A)**. Imaging and laboratory examinations of the patient. In March 2021, CT scan showed bilateral pneumonia, pleural and pericardial effusion (arrow) **(B,C)**; CT scans in October 2022 indicated left lower atelectasis **(D)**. CT scan in June 2023 showed significant improvement in both lungs **(E)**. The neutralizing activity by comparing pSTAT1 in the patient to that of controls using flow cytometry [CD14 + cells in PBMCs isolated form a healthy volunteer were incubated with IFN-γ (10 ng/ml) in the presence of 2% patient's plasma **(F)**. Assessment of plasma concentrations of AIGAs by enzyme-linked immunosorbent assay and evaluation of inhibition of STAT-1 phosphorylation during follow-up, Median fuorescent intensity (MFI) from each sample from diferent groups was calculated as % pSTAT-1 MFI stimulation index = [(MFIplasma + IFN-γ—MFIunstimulated/MFIIFN-γ—MFIunstimulated) × 100] **(G)**.

A 13-year-old HIV-negative Chinese boy presented to the hospital with a seven-month history of fever, cough, and enlarged cervical lymph nodes, in March 2021 (clinical course in Figure 1A).

He and his older sister were born to healthy and non-consanguineous parents, and had no history of infection, autoimmune diseases, autoinflammatory conditions, allergies, or malignancies. Initially suspected of having a Mycobacterium tuberculosis infection by the local hospital, he received a combination of clarithromycin, clindamycin, isoniazid, rifampicin, pyrazinamide, and ethambutol. However, his condition showed little improvement. Laboratory tests revealed leukocytosis (31 × 10^9^/L) with neutrophilia (85.3%), anemia (hemoglobin: 83 g/L), thrombocytosis (596 × 10^9^/L), eosinophilia (0.5 × 10^9^/L). Inflammatory markers were elevated, including C-reactive protein (11 mg/dl) and erythrocyte sedimentation rate (68 mm/h). Serum immunoglobulin levels showed elevated IgG (23.02 g/L) and IgE (175.5 IU/ml). Flow cytometry assay revealed mildly elevated CD4 T cells at 1,173 cells/µl, CD8 T cells at 1,187 cells/µl, B cells at 650 cells/µl, but mildly reduced NK cell at 166 cells/µl. A computed tomography (CT) scan showed bilateral pneumonia, pleural and pericardial effusions (Figures 1B,C). Notably, blood culture yielded *Mycobacterium abscessus*, and TM grew on the culture from pericardium effusion*.* The diagnosis of disseminated co-infections with TM and *M. abscessus* was made.

Disseminated co-infections with NTM and TM are rare in immunocompetent individuals. In pediatric patient, primary immunodeficiencies (PID) are the common cause of opportunity infections. Therefore, whole exome sequencing (WES) was performed and it unveiled that no pathogenic variants were found. Surprisingly, the result of QuantiFERON-TB Gold InTube test was indeterminate (negative control < 2 pg/ml; positive control < 2 pg/ml; TB antigen tube < 0 pg/ml), and the indeterminate result was previously reported in patients suffering NTM infection with AIGAs (10). Considering AIGAs are the major cause of disseminated TM or NTM infection in non-HIV infected patients from southeast Asia, AIGAs screen was conducted and high concentrations of AIGAs was identified [94.5 μg/ml, well beyond the range of healthy controls (<2 μg/ml)] in his serum, which effectively blocked IFN-γ-induced signal transducer and activator of transcription-1 (STAT-1) phosphorylation in healthy control's peripheral blood mononuclear cells, comfirming the functional neutralization of AIGAs (Figure 1F). The patient's human leukocyte antigen typing revealed DRB1*08:03, DRB1*16:02, DQB1*05:02, and DQB1*06:01. Following the above diagnosis, the patient commenced treatment with oral azithromycin (0.45 g qd) and linezolid (0.6 g qd), intravenous amikacin (0.4 g qd) for *M. abscessus* infection, and oral voriconazole (0.2 g bid) for TM infection. Subsequently, his symptoms resolved, and a chest CT scan conducted 4 months later showed significant improvement, leading to the discontinuation of azithromycin and amikacin. However, in March 2022, symptoms of fever and cough recurred, and a CT scan showed left lower atelectasis in October 2022 (Figure 1D). Metagenomic next-generation sequencing of bronchoalveolar lavage fluid revealed the presence of *Mycobacterium asiaticum*. Consequently, oral azithromycin (0.5 g qd) was reintroduced. In June 2023, Voriconazole was discontinued as the disease had entered remission (Figure 1E). The patient is in stable condition and returns to our clinic regularly for follow-up. Over time, the plasma concentrations of AIGAs decreased, and their neutralizing ability also decreased (Figure 1G).

## 3 Discussion

In this report, we present a rare case of an adolescent with AIGAs who experienced TM and multiple NTM infections. To the best of our knowledge, no such patient has ever been documented in the literature.

In HIV-negative children, disseminated infections of NTM or TM are often observed in those with secondary immunodeficiency due to prolonged use of immunosuppressive drugs, organ transplantations, or malignancies. Additionally, PID resulting from mutations in various genes can also increase susceptibility to these infections in affected individuals (11–13). Considering the patient's narrow-spectrum infectious susceptibility to TM and NTM, an NGS panel targeting genes associated with inborn errors of innate and intrinsic immunity should be prioritized over WES, as it is more cost-effective and offers faster testing and diagnosis (14). Given the narrow-spectrum infectious susceptibility to pathogens, the substantial normality of standard lymphocyte typing, the absence of pathogenic variants at WES, and the absence of a relevant history of infectious susceptibility and immunodysregulation, the patient's overall picture could be framed as a phenocopy of inborn errors of innate and intrinsic immunity, usually affecting otherwise healthy patients, whose genetic counterpart is the inborn errors of innate and intrinsic immunity belonging to category VI of the IUIS (7). Therefore, these evidence led us to suspect the presence of AIGAs. Notably, we confirmed neutralizing AIGAs, which is responsible for the TM and NTM infection in the proband. Thus, a first, or recurrent episodes of, unexplained life-theatening TM or NTM infection in otherwise healthy children should raise suspicion of an underlying IEI, especially the presence of AIGAs (15). We propose that routine AIGAs testing should be considered in pediatric patients presenting with opportunistic infections, particulary those involving TM or NTM infections. In certain diseases, such as delayed onset combined immunodeficiency with granulomas and/or autoimmunity due to RAG1 mutation, common variable immunodificiency, and etc, AIGAs could be detected. However, AIGAs in those conditions usually have no neutralizing ability (1, 16). Un-In Wu et al. have reported QuantiFERON Gold In-Tube test could be used as an alternative screening method for AIGAs due to its widespread availability and clinical accessibility (10). In patients with AIGAs, these autoantibodies can neutralize IFN-γ secretion during the mitogen stimulation in the test, resulting in an indeterminate result. An indeterminate result suggests the presence of AIGAs. However, indeterminate results are not exclusive to AIGAs patients. They can also occur in individuals with other conditions, such as those with MSMDs or patients on immunosuppressive therapies (17, 18).

Reported AIGAs cases have predominantly reported in individuals of Southeast Asia. And the production of AIGAs may be associated with high-risk HLA alleles, such as HLA-DRB1*15:02/16:02 and HLA-DQB1*05:01/05:02 (19). Consistent with previous studies, this pediatric patient also carried the high-risk HLAs DRB1*16:02 and DQB1*05:02. However, his parents tested negative for AIGAs, suggesting other unidentified factors might be involved in the production of AIGAs. Among over 600 previously reported AIGAs cases, only two were under 18 years old, both originating from Southeast Asia, and both experiencing refractory NTM infection (20). Our patient initially experienced co-infetions with TM and *M. abscessus*. He discontinued azithromycin and amikacin after 4 months due to clinical improvement but continued to suffer from *M.asiaticum* infection even after 19 months. This may be attributable to the persistent immunodeficient state caused by the potent neutralizing effect of AIGAs, even though the AIGAs titer had decreased. This highlights the necessity of prolonged antimicrobial therapy and continuous monitoring of both AIGAs levels and their neutralizing capacity in patients with AIGAs. However, there were no established guidelines about when to discontinue antimicrobial treatment. Despite long-term and intensive antimicrobial treatments, more than half of patients with AIGAs experienced a persistent or relapsed infection (21). Adjunctive immunomudulatory treatments targeting T cells or B cells, such as cyclophosphamide, rituximab, bortezomib, abatacept, and daratumumab have been reported to be associated with favorable clinical outcomes in severe or refractory cases (22). However, these adjunctive treatments were based on scattered case and may increase risk of infections so the patients should be closely monitored during treatment. Importantly, further clinical trials are needed to better assess their safety and efficacy.

In summary, this case emphasizes the importance of screening for AIGAs in pediatric patients with TM or NTM infections. In patients with AIGAs, attention should be paid to the potential of co-infection or subsequent infections with emerging pathogens, including TM, NTM and *Salmonella spp.* Treatment of these patients remains challenging, and prolonged antimicrobial therapy is crucial. While adjunctive treatments aimed at reducing AIGAs production have shown partial success in severe or refractory cases, further clinical studies are still needed to better evaluate their effectiveness.

## Data Availability

The raw data supporting the conclusions of this article will be made available by the authors, without undue reservation.

## References

[1] Cheng A Holland SM. Anti-cytokine autoantibodies: mechanistic insights and disease associations. Nat Rev Immunol. (2024) 24(3):161–77. 10.1038/s41577-023-00933-2 37726402

[2] Arts RJW Janssen NAF van de Veerdonk FL. Anticytokine autoantibodies in infectious diseases: a practical overview. Int J Mol Sci. (2023) 25(1):515. 10.3390/ijms25010515 38203686 PMC10778971

[3] Vignali DA Kuchroo VK. IL-12 family cytokines: immunological playmakers. Nat Immunol. (2012) 13(8):722–8. 10.1038/ni.2366 22814351 PMC4158817

[4] Casanova JL MacMicking JD Nathan CF. Interferon-**γ** and infectious diseases: lessons and prospects. Science. (2024) 384(6693):eadl2016. 10.1126/science.adl2016 38635718 PMC12539790

[5] Yang R Mele F Worley L Langlais D Rosain J Benhsaien I Human T-bet governs innate and innate-like adaptive IFN-γ immunity against mycobacteria. Cell. (2020) 183(7):1826–1847.e31. 10.1016/j.cell.2020.10.046 33296702 PMC7770098

[6] Puel A Bastard P Bustamante J Casanova JL. Human autoantibodies underlying infectious diseases. J Exp Med. (2022) 219(4):e20211387. 10.1084/jem.20211387 35319722 PMC8952682

[7] Bousfiha A Moundir A Tangye SG Picard C Jeddane L Al-Herz W The 2022 update of IUIS phenotypical classification for human inborn errors of immunity. J Clin Immunol. (2022) 42(7):1508–20. 10.1007/s10875-022-01352-z 36198931

[8] Shih HP Ding JY Yeh CF Chi CY Ku CL. Anti-interferon-γ autoantibody-associated immunodeficiency. Curr Opin Immunol. (2021) 72:206–14. 10.1016/j.coi.2021.05.007 34175547

[9] Guo J Ning XQ Ding JY Zheng YQ Shi NN Wu FY Anti-IFN-γ autoantibodies underlie disseminated *Talaromyces marneffei* infections. J Exp Med. (2020) 217:e20190502. 10.1084/jem.20190502 32880631 PMC7953730

[10] Wu UI Chuang YC Sheng WH Sun HY Jhong YT Wang JY Use of QuantiFERON-TB gold in-tube assay in screening for neutralizing anti-interferon-γ autoantibodies in patients with disseminated nontuberculous mycobacterial infection. Clin Microbiol Infect. (2018) 24(2):159–65. 10.1016/j.cmi.2017.06.029 28694201

[11] Meoli A Deolmi M Iannarella R Esposito S. Non-tuberculous mycobacterial diseases in children. Pathogens. (2020) 9(7):553. 10.3390/pathogens9070553 32660053 PMC7400539

[12] Bustamante J. Mendelian Susceptibility to mycobacterial disease: recent discoveries. Hum Genet. (2020) 139(6-7):993–1000. 10.1007/s00439-020-02120-y 32025907 PMC7275902

[13] Qiu Y Feng X Zeng W Zhang H Zhang J. Immunodeficiency disease spectrum in HIV-negative individuals with *Talaromycosis*. J Clin Immunol. (2021) 41(1):221–3. 10.1007/s10875-020-00869-5 32996007

[14] Cifaldi C Brigida I Barzaghi F Zoccolillo M Ferradini V Petricone D Targeted NGS platforms for genetic screening and gene discovery in primary immunodeficiencies. Front Immunol. (2019) 10:316. 10.3389/fimmu.2019.00316 31031743 PMC6470723

[15] Moratti M Conti F Giannella M Ferrari S Borghesi A. How to: diagnose inborn errors of intrinsic and innate immunity to viral, bacterial, mycobacterial, and fungal infections. Clin Microbiol Infect. (2022) 28(11):1441–8. 10.1016/j.cmi.2022.07.021 35934195

[16] Walter JE Rosen LB Csomos K Rosenberg JM Mathew D Keszei M Broad-spectrum antibodies against self-antigens and cytokines in RAG deficiency. J Clin Invest. (2016) 126(11):4389. 10.1172/JCI91162 27801680 PMC5096894

[17] Bellagha R Dhaouadi T Riahi A Ben Rehouma W Jedidi H Mouelhi L A regression predictive model for QuantiFERON-TB gold Plus® indeterminate results in immunosuppressed patients. SAGE Open Med. (2024) 12:20503121241279116. 10.1177/20503121241279116 39263635 PMC11388302

[18] Dalvi A Bargir UA Natraj G Shah I Madkaikar M. Diagnosis and management of infections in patients with mendelian susceptibility to mycobacterial disease. Pathogens. (2024) 13(3):203. 10.3390/pathogens13030203 38535546 PMC10975294

[19] Pithukpakorn M Roothumnong E Angkasekwinai N Suktitipat B Assawamakin A Luangwedchakarn V HLA-DRB1 and HLA-DQB1 are associated with adult-onset immunodeficiency with acquired anti-interferon-γ autoantibodies. PLoS One. (2015) 10:e0128481. 10.1371/journal.pone.0128481 26011559 PMC4444022

[20] Liew WK Thoon KC Chong CY Tan NWH Cheng DT Chan BSW Juvenile-onset immunodeficiency secondary to anti-interferon-γ autoantibodies. J Clin Immunol. (2019) 39(5):512–8. 10.1007/s10875-019-00652-1 31177358

[21] Chi CY Lin CH Ho MW Ding JY Huang WC Shih HP Clinical manifestations, course, and outcome of patients with neutralizing anti-interferon-γ autoantibodies and disseminated nontuberculous mycobacterial infections. Medicine. (2016) 95(25):e3927. 10.1097/MD.0000000000003927 27336882 PMC4998320

[22] Cheng A Holland SM. Anticytokine autoantibodies: autoimmunity trespassing on antimicrobial immunity. J Allergy Clin Immunol. (2022) 149(1):24–8. 10.1016/j.jaci.2021.11.016 34998474 PMC9034745

